# Importation of Dengue Virus Type 3 to Japan from Tanzania and Côte d’Ivoire

**DOI:** 10.3201/eid1611.101061

**Published:** 2010-11

**Authors:** Meng Ling Moi, Tomohiko Takasaki, Akira Kotaki, Shigeru Tajima, Chang-Kweng Lim, Mitsuo Sakamoto, Hajime Iwagoe, Kenichiro Kobayashi, Ichiro Kurane

**Affiliations:** Author affiliations: National Institute of Infectious Diseases, Tokyo, Japan (M.L. Moi, T. Takasaki, A. Kotaki, S. Tajima, C.-K. Lim, I. Kurane);; Kawasaki Municipal Kawasaki Hospital, Kanagawa, Japan (M. Sakamoto);; Kumamoto City Hospital, Kumamoto City, Japan (H. Iwagoe);; Tokyo Metropolitan Bokutoh Hospital, Tokyo (K. Kobayashi)

**Keywords:** Dengue, Tanzania, Côte d’Ivoire, viruses, vector-borne infections, dispatch

## Abstract

Travelers can introduce viruses from disease-endemic to non–disease-endemic areas. Serologic and virologic tests confirmed dengue virus infections in 3 travelers returning to Japan: 2 from Tanzania and 1 from Côte d’Ivoire. Phylogenetic analysis of the envelope gene showed that 2 genetically related virus isolates belonged to dengue virus type 3 genotype III.

Dengue virus (DENV) is an arthropod-borne virus that infects ≈100 million persons each year in Southeast Asia, Central and South America, and Africa. Infection with any 1 of the 4 DENV serotypes causes a wide range of disease, from dengue fever to the more severe dengue hemorrhagic fever. Epidemics of dengue-like illness have occurred in Africa, but information about etiology is limited (*1*). Transmission of another arthropod-borne virus, chikungunya virus, which causes disease similar to dengue, has been documented in Tanzania. Although Tanzania is on the list of countries at risk for DENV transmission (*2*), to our knowledge, no DENV isolates have yet been identified and studied there.

In Japan as of June 16, 2010, a total of 50 cases of imported dengue had been reported. Among these cases, 2 were dengue fever that had developed in 2 travelers after they returned from Tanzania. We report the molecular characterization of 2 DENV type 3 (DENV-3) isolates from 1 of the travelers who had visited Tanzania in 2010 and from a traveler who had visited Côte d’Ivoire in 2008.

## The Study

In 2010, a 55-year-old man (patient 1) and a 23-year-old woman (patient 2) returned to Japan from Tanzania; high fever and thrombocytopenia developed in each on days 1 and 3 days after return, respectively. In 2008, a 65-year-old man (patient 3) returned to Japan from Côte d’Ivoire and subsequently experienced high fever. Serum samples from each of the 3 patients were sent to the National Institute of Infectious Diseases, Japan, for laboratory examination. DENV serotypes were determined by serotype-specific reverse transcription–PCR (RT-PCR) (*3*). DENV-specific immunoglobulin (Ig) M was detected by Dengue Fever Virus IgM Capture ELISA (Focus Diagnostics, Inc., Cypress, CA, USA) used according to the manufacturer’s instructions. Dengue IgG Indirect ELISA (Panbio Ltd, Sinnamon Park, Queensland, Australia) was used to detect anti-DENV IgG according to the manufacturer’s instructions. Serum from patient 1 was negative for anti-DENV IgM and anti-DENV IgG; serum from patients 2 and 3 was positive for anti-DENV IgM and IgG. All 3 serum samples had positive DENV nonstructural protein (NS) 1 antigen results according to NS1 capture ELISA (Platelia Dengue NS1 Antigen assay; Bio-Rad Laboratories, Marnes-la-Coquette, France) and negative chikungunya viral RNA results by RT-PCR.

DENV-3 (D3/Hu/Tanzania/NIID08/2010) was isolated from patient 1, and DENV-3 (D3/Hu/Côte d’Ivoire/NIID48/2008) was isolated from patient 3 by using the *Aedes albopictus* mosquito cell line C6/36 and FcγR-expressing baby hamster kidney cells (*4*). DENV-3 RNA was detected in serum from patient 2 by RT-PCR, but the virus was not isolated. The viral RNA was extracted by using a High Pure Viral RNA Extraction kit (Roche Diagnostics, Mannheim, Germany), transcribed to cDNA, amplified by PCR, and sequenced as described (*3*).

Nucleotide sequences of the isolates were compared with selected sequences of DENV-3 (Table). Sequence alignment and phylogenetic analysis was performed by the Genetyx analysis program (Genetyx Corp., Tokyo, Japan). The phylogenetic tree was constructed by using the neighbor-joining method. The selected DENV-3 strains were grouped into 5 genotypes (*5*). Confidence values for virus groupings were assessed by bootstrap assembling analysis of 1,000 replicates. The 2 DENV-3 isolates belonged to DENV-3 genotype III (Figure). The envelope (E)-protein sequence showed that the DENV-3 (D3/Hu/Tanzania/NIID08/2010 strain) isolated from patient 1 had a sequence homology of 98% to the DENV-3 D3/Hu/Côte d’Ivoire/NIID48/2008 strain and 99% to a DENV-3 6805 strain isolated in Saudi Arabia in 2004 (GenBank accession no. AM746229) (*6*).

**Table:** Comparison of dengue virus type 3 sequences from travelers returning from Tanzania and Côte d’Ivoire with selected dengue virus type 3 sequences*

Year isolated	Name	Strain	Isolate origin	GenBank accession no.
2010	Tanzania2010	D3/Hu/Tanzania/NIID08/2010	Tanzania	AB549332
2008	Côte d’Ivoire2008–1	D3/Hu/Côte d'Ivoire/NIID48/2008	Côte d’Ivoire	AB447989
2008	Côte d’Ivoire2008–2	2008/00510	Côte d’Ivoire	FM213456
2007	Bhutan2007–1	SV0786_07	Bhutan	FJ606712
2007	Bhutan2007–2	SV0837_07	Bhutan	FJ606708
2004	Saudi Arabia2004–1	6805	Saudi Arabia	AM746229
2004	Saudi Arabia2004–2	6475	Saudi Arabia	AM746232
2004	Colombia2004	22379_MEDELLIN/04	Colombia	FJ389910
2003	India2003	GWL-25	India	AY770511
2001	Venezuela2001	LARD6667	Venezuela	AY146773
2000	Brazil2000	68784	Brazil	AY038605
2000	Venezuela2000	LARD6315	Venezuela	AY146767
2000	Cambodia–India2000†	00–28–1HuNIID	Cambodia/India	AB111081
1997	Thailand1997	D97–0106	Thailand	AY145728
1995	Honduras1995	HN179	Honduras	FJ189469
1992–1994	Malaysia1992–1994–1	LN1746	Malaysia	AF147458
1992–1994	Malaysia1992–1994–2	LN2632	Malaysia	AF147459
1992	Fiji1992	29472	Fiji	L11422
1991	Sri Lanka1991	2783	Sri Lanka	L11438
1990	Sri Lanka1990	SK698	Sri Lanka	FJ189449
1989	Tahiti1989	2167	Tahiti	L11619
1989	Sri Lanka1989	260698	Sri Lanka	L11437
1986	Thailand1986	D86–007	Thailand	L11441
1985	Mozambique1985	1559	Mozambique	L11430
1984	India1984	1416	India	L11424
1983	Philippines1983	168.AP-2	Philippines	L11432
1981	Malaysia1981	29586	Malaysia	L11427
1963	Puerto Rico1963–1	PR6	Puerto Rico	L11433
1963	Puerto Rico1963–2	BS-PRico63	Puerto Rico	AY146762
1956	Philippines1956	H87	Philippines	L11423
2001	DENV-1	01–44–1HuNIID	Tahiti	AB111070
2005	DENV-2	D2/Hu/OPD030NIID/2005	East Timor	AB219135
2001	DENV-4	MY01–22713	Malaysia	AJ428556

*DENV, dengue virus. †Strain 00–28–1HuNIID was isolated from a traveler who returned to Japan after visiting Cambodia and India.

**Figure:**
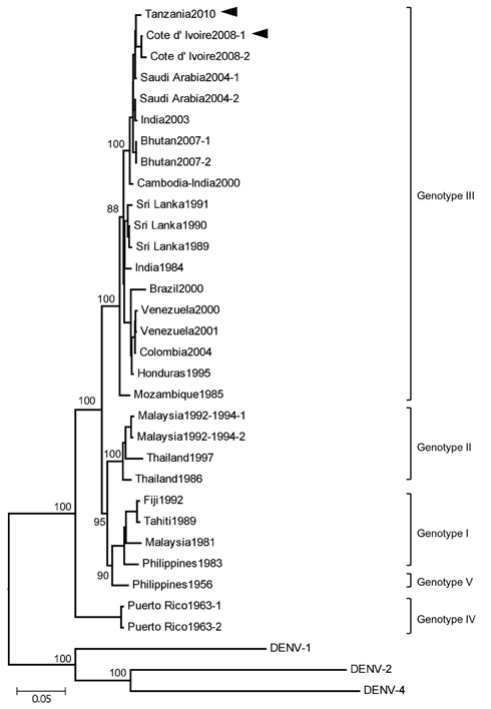
Phylogenetic tree based on the envelope genome sequence of selected dengue virus type 3 (DENV-3) strains. The tree was rooted to DENV-1, DENV-2 and DENV-4. Multiple sequence alignments were performed, and the tree was constructed by using the neighbor-joining method. The percentage of successful bootstrap replication is indicated at the nodes. DENV-3 genotypes are indicated on the right. The isolated DENV-3 strains, D3/Hu/Tanzania/NIID08/2010 strain (Tanzania2010) and D3/Hu/Côte d'Ivoire/NIID48/2008 strain (Côte d’Ivoire2008), are indicated with arrowheads. Scale bar indicates nucleotide substitutions per site.

## Conclusions

DENV transmission has occurred in western Africa and some parts of eastern Africa (*7*–*10*). We isolated DENV-3 from 2 patients in Japan in whom dengue fever developed after they returned from Côte d’Ivoire (western Africa) and Tanzania (eastern Africa). Detection of DENV-3 in patients 1 and 2 suggests local DENV-3 transmission in Tanzania. As of April 2010, at least 17 suspected cases of dengue have been reported among residents of Dar es Salaam, Tanzania, but the molecular epidemiology of DENV in Tanzania has not been analyzed (*11*). In the absence of such analyses, data on molecular epidemiology of DENV isolated from returning travelers offers timely information to countries where dengue surveillance is not routinely performed. Data and reports of the presence of a competent DENV vector, *Ae. aegypti* mosquitoes, suggest the need for further studies on local DENV transmission in Tanzania (*12*).

It is assumed that travelers increase the risk for introduction of DENV serotypes or strains from disease-endemic to non–disease-endemic areas where competent vectors such as *Ae. aegypti* or *Ae. albopictus* mosquitoes are present. The sequence homology among the DENV-3 strain isolated from the traveler to Tanzania (patient 1), the DENV-3 strain isolated from the traveler from Côte d’Ivoire in 2008 (patient 3), and a DENV-3 strain isolated in Saudi Arabia in 2004 ranged from 98% to 99% (GenBank accession nos. AB549332, AB447989, AM746229, respectively). Because the E-protein gene of the isolate from Tanzania was highly similar to those in viruses circulating regionally and the Middle East, the disease could have been introduced or reintroduced into the country from neighboring areas. These data suggest the need for further studies of the route of disease dissemination and surveillance of dengue in Africa. Genetic differences among subtypes may result in differences in virus virulence and epidemic potential (*13*). DENV-3 genotype III, previously isolated from several parts of Africa, Latin America, and the Indian subcontinent, has been associated with higher incidence of major epidemics of dengue and dengue hemorrhagic fever (*14*). The DENV isolates from Tanzania and Côte d’ Ivoire were closely related to a DENV-3 genotype III strain isolated from a major DENV outbreak in northern India in 2003–2004; the E-protein gene homology was 98% (*15*).

Emergence of DENV-3 genotype III in geographically diverse areas may thus result from higher epidemic potential of the virus, although further studies are needed to understand the clinical and epidemiologic implications of emergence or reemergence of the virus in Tanzania and Côte d’ Ivoire. Whereas other vector-borne diseases such as malaria and yellow fever have been well studied in Africa, dengue needs more attention with regard to identification of epidemics, clinical implications, and disease management.
